# MRI radiomics-based approach to predict pituitary neuroendocrine tumor invasiveness

**DOI:** 10.1186/s41747-026-00736-9

**Published:** 2026-05-20

**Authors:** Rosalinda Calandrelli, Huong Elena Tran, Edda Boccia, Elia Oliva, Gabriella D’Apolito, Luca Boldrini, Pier Paolo Mattogno, Sabrina Chiloiro, Marco Gessi, Francesco Doglietto, Simona Gaudino

**Affiliations:** 1 Dipartimento di Diagnostica per Immagini e Radioterapia Oncologica, Fondazione Policlinico Universitario “A. Gemelli” IRCCS, Rome, Italy; 2 Radiomics GSTeP Core Research Facility, Fondazione Policlinico Universitario “A. Gemelli” IRCCS, Rome, Italy; 3 Università Cattolica del Sacro Cuore, Rome, Italy; 4 Neurosurgery, Dipartimento di neuroscienze, Organi di Senso e Torace, Fondazione Policlinico Universitario “A. Gemelli” IRCCS, Rome, Italy; 5 Endocrinology, Fondazione Policlinico Universitario “A. Gemelli” IRCCS, Rome, Italy; 6 Pathology, Fondazione Policlinico Universitario “A. Gemelli” IRCCS, Rome, Italy

**Keywords:** Magnetic resonance imaging, Machine learning, Neuroendocrine tumors, Pituitary gland, Radiomics

## Abstract

**Objectives:**

To assess the diagnostic potential of magnetic resonance imaging (MRI) radiomics and machine learning models using T2-weighted and contrast-enhanced (CE)-T1-weighted images, individually and combined, to predict the invasiveness of pituitary neuroendocrine tumors (PitNETs).

**Materials and methods:**

Patients with macro-PitNETs were retrospectively enrolled from 2019 to 2022. Radiomic features were extracted from manually segmented lesions on preoperative T2-weighted and CE-T1-weighted images and, after a feature selection step, used to assess invasiveness, defined following Trouillas’ classification. Five machine learning models (logistic regression, random forest, gradient boosting, AdaBoost, XGBoost) were trained using CE-T1-weighted, T2-weighted, and CE-T1-weighted plus T2-weighted features. Performance was evaluated on a test set using the area under the receiver operating characteristic curve (AUC).

**Results:**

Two hundred patients were included in the study: 95 PitNETs were noninvasive (74 grade 1a; 21 grade 1b) and 105 invasive (70 grade 2a; 35 grade 2b). A total of 102 radiomic features were extracted per sequence. The best-performing model was the XGBoost, using five combined CE-T1-weighted and T2-weighted features, with an AUC of 0.85 (95% confidence interval: 0.75‒0.95). Lower AUC values were obtained for logistic regression using CE-T1-weighted images (0.80) and AdaBoost using T2-weighted images (0.78).

**Conclusion:**

The XGBoost model, incorporating tumor shape, texture, and first-order features extracted from both CE-T1-weighted and T2-weighted MRI, showed high performance in predicting PitNETs invasiveness. This radiomic model might help identify tumors with a higher risk of disease persistence, recurrence, or progression.

**Relevance statement:**

The radiomic model based on contrast-enhanced T1-weighted and T2-weighted MRI demonstrated high discriminative ability in predicting invasiveness of pituitary neuroendocrine tumors and could aid in identifying tumors that may be at higher risk for recurrence or progression, ultimately improving patient outcomes through personalized treatment strategies.

**Key Points:**

- Pituitary neuroendocrine tumors (PitNETs) represent a significant challenge in clinical practice.
- Accurate preoperative prediction of PitNET invasiveness is crucial for surgery and prognosis.
- Contrast-enhanced T1-weighted and T2-weighted MRI-based radiomic model effectively predicts PitNET invasiveness.
- The developed radiomic model could help optimize individualized treatment decisions before surgery.

**Graphical Abstract:**

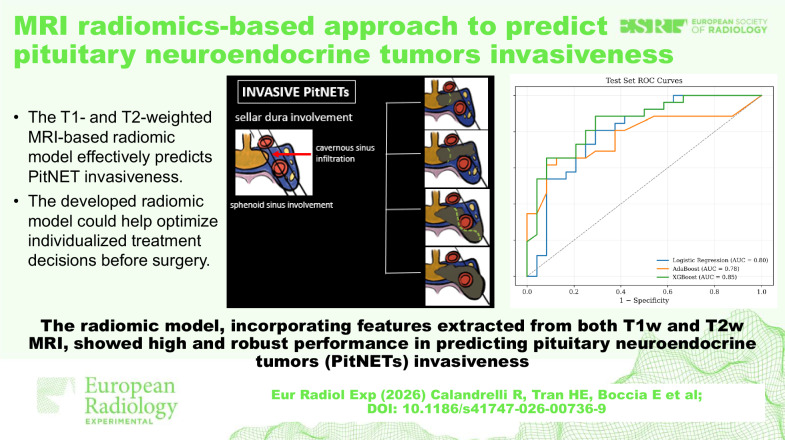

## Background

Pituitary neuroendocrine tumors (PitNETs) correspond to 10–25% of intracranial tumors, with 17–20% prevalence in the general population [1, 2]. Despite often being benign, 25–55% of PitNETs show invasive growth [1, 2], and 10–20% recur after resection [3]. Macro-PitNETs are characterized by large size and extension into surrounding structures [4]. “Invasiveness” refers to PitNETs with infiltrative growth into surrounding structures observed during surgery, such as the dura, bones, and rarely nerves and vessels [4], while “aggressiveness” refers to rapid growth, invasion capability, and resistance to treatment, requiring prolonged follow-up [5–7]. Several studies demonstrated that invasiveness, particularly dural invasiveness, significantly influences surgical outcome and prognosis [8].

The most recently proposed classification system for predicting disease recurrence or persistence is Trouillas’ classification system, scoring PitNETs into five grades [9]. This combined clinicopathological classification overcomes the limitations of using only the radiological assessment of tumor invasiveness and aggressiveness. Knosp’s grading system (grades 1‒4) is the most widely accepted preoperative radiological method to assess parasellar extension in PitNETs; however, it may fail to accurately predict true cavernous sinus invasion in intermediate grades (2 or 3), as distinguishing between invasion and compression remains challenging in radiological imaging [10, 11]. According to the 2022 World Health Organization classification, certain subtypes of pituitary adenomas (silent corticotrophs, sparsely granulated growth hormone PitNETs, lactotrophs in men, Crooke cell PitNETs, mature plurihormonal pituitary‑specific positive transcription factor (PIT)-1 lineage tumors, immature PIT-1 lineage, acidophil stem cell tumors) are “high-risk” tumors and could be classified as “stage b” in a revised version of Trouillas’ criteria [12].

Surgical removal is the first-line treatment for most pituitary macro-PitNETs [13, 14], though challenges remain in achieving complete removal, especially in invasive cases [15–18].

Accurate preoperative prediction of PitNETs’ invasiveness is essential for effective surgical strategy, personalized follow-up and long-term management.

MRI represents the best imaging technique for defining the tumor’s anatomical relationships with surrounding structures [19]. Radiomics is an innovative advancement in quantitative bioimaging analysis based on extracting a vast array of information that describes the shape and texture of tumors from radiological images, translating them into numerical features [20–22]. These features capture subtle patterns and heterogeneity within the tumor that may not be easily discernible through visual inspection alone, offering insights into predicting tumor subtypes and prognosis [23]. Machine learning (ML), a branch of artificial intelligence, develops predictive models to enhance diagnostic and prognostic accuracy [24].

To date, radiomics studies of PitNET have predominantly focused on predicting specific clinicopathological characteristics of the tumor, including tumor subtypes, consistency, proliferation indices, and cavernous sinus invasiveness [25–29]. Meanwhile, few studies have investigated the capability of an MRI-based radiomics-clinicopathological model to predict invasiveness according to Trouillas’ final grading system.

This study aimed to: (1) classify macro-PitNETs using Trouillas’ radiological and histological criteria, including “high-risk” adenomas in the proliferative tumor class; (2) assess the association between MRI-based radiomic features and the final tumor grade; (3) evaluate the diagnostic potential of preoperative MRI radiomics features and machine learning models, built on T2-weighted and contrast-enhanced (CE) T1-weighted images, individually and in combination, for predicting PitNET invasiveness classified according to Trouillas’ grading system.

## Methods

The CLEAR/CLEAR-E3 guidelines were followed, and the checklist is reported in the Supplementary material (Supplementary Table S1).

### Dataset description

#### Patient selection

This observational non-interventional retrospective study was approved by our institutional ethics committee (ID 5846). Clinical and imaging data of patients undergoing their first surgery for PitNETs at Fondazione Policlinico Universitario “A. Gemelli” IRCCS from September 2019 to February 2022 were recorded. Inclusion criteria: patients with macro-PitNETs, pathologically confirmed, and a complete MRI protocol. Exclusion criteria: previous PitNET treatments (surgical, medical, or radiation therapies); PitNETs with significant cystic or hemorrhagic components.

#### Surgical technique and findings

Patients underwent endoscopic transsphenoidal surgery. Intraoperative invasiveness of the cavernous sinus, dural sellar floor, and sphenoid sinus was evaluated. Specimens of suspected invaded tissue (sellar periosteum, medial and anterior wall of the cavernous sinus, sphenoid mucosa) were collected for histological examination.

#### Clinical features

For each patient, medical history, baseline radiological characteristics, and complete hormonal testing were recorded. Patients were classified according to: (1) tumor volume; (2) endocrinological status and tumor histotypes according to guidelines [30, 31]; (3) histopathological classification into three cell lineages: PIT-1, steroidogenic factor-1 (SF-1), and T‑box pituitary transcription factor, TBX19 (T)-PIT transcription factor; null-cell adenomas and high-risk adenomas were also characterized [31–33]; (4) tumor invasiveness: Knosp grade 3 or 4 for the cavernous sinus invasion on MRI, Knosp grade 1 or 2 with invasiveness evidence of the medial wall of the cavernous sinus, the dura mater of the sella and the sphenoid sinus, histologically and intraoperatively confirmed; (5) proliferative status as reported in previous studies [16, 34]; “High-risk” PitNETs were considered proliferative tumors.

#### Final grading system score and tumor categorization

PitNETs were classified into five Trouillas grades including high-risk tumors in the proliferative class. Noninvasive (grades 1a and 1b) and invasive (grades 2a and 2b) tumors were classified as Category 1 and 2, respectively.

#### Image acquisition

MRI images (acquired within 6 months pre-surgery) were obtained using 1.5-T scanners (Signa units (General Electric Healthcare) and Philips Ingenia (Philips Healthcare)) with an eight-channel head coil. The imaging protocol included coronal T1 and T2-weighted sequences, axial and coronal CE-T1-weighted sequences with slice thickness of 2.5 mm. CE-T1-weighted images were acquired 2‒4 min after injection of 0.1 mmol/kg of gadobutrol (Gadavist; Bayer Schering Pharma) at 2 mL/s *via* antecubital venous access.

#### Image segmentation

Coronal T2-weighted and CE-T1-weighted images were used to segment the lesion slice-by-slice, manually outlining the volume of interest while excluding surrounding elements such as cerebrospinal fluid, bone, and calcifications within the tumor. Segmentation was performed using the open-source software ITK-SNAP (version 4.0.0, Kitware), applying the Cavalieri principle [35, 36]. A neuroradiologist with 10 years of experience performed segmentation, reviewed by a second neuroradiologist to ensure consistency among measurements. Both were blinded to intraoperative and clinical data.

### Radiomics analysis

#### Image pre-processing

Bias-field correction addressed low-frequency intensity artifacts [37]. A rigid transformation aligned T2-weighted segmentations with CE-T1-weighted images using nearest-neighbor resampling [38]. Resampling to the median value of the pixel spacing distribution (*i.e*., 0.45 mm) ensured uniform in-plane resolutions, and *z*-score normalization was performed per image.

#### Radiomic feature extraction

Features were extracted from CE-T1-weighted and T2-weighted volumes of interest using Pyradiomics (version 3.1.0) [39] and divided into three families: (1) morphological features describing tumor shape and size; (2) first-order features reflecting tumor global gray intensity; (3) texture features capturing tumor local gray intensity, extracted with a bin count of 70 [40] and divided into: gray-level cooccurrence matrix (GLCM); gray-level run-length matrix (GLRLM); gray-level size zone matrix (GLSZM); gray-level dependence matrix (GLDM) [41].

Due to out-of-plane resolution variability, features were extracted in two-dimensional (2D) mode along the coronal plane throughout the entire volume (setting force2D = True). All other parameters remained as a default configuration.

#### Study design for radiomics modeling

ML models were developed to predict the invasiveness of solid or microcystic PitNETs. Three analyses were conducted using features from CE-T1-weighted images, T2-weighted images, and both sequences. For each of the three analyses (CE-T1-weighted, T2-weighted, CE-T1-weighted + T2-weighted), five ML models were implemented using the Python library scikit-learn: logistic regression, random forest, gradient boosting, AdaBoost, and XGBoost.

The dataset was split (75% training, 25% testing for internal validation), ensuring consistent proportions of invasive tumors. The *z*-score normalization was applied to radiomic features of the training set, and the parameters found for scaling were used in the test set. These models took selected features as input and output the probability of macro-PitNET invasiveness, with a threshold of 0.5 for binary classification.

### Statistical analysis and modeling

Statistical analysis and modeling were performed in RStudio (R version 3.4.1) and Python (v3.7).

#### Clinical characteristics

Descriptive statistics were expressed as median and interquartile range for continuous variables and as numbers/percentages for qualitative variables. The Shapiro–Wilk test assessed data normality. The Kruskal–Wallis test compared age across the final grading system’s grades and tumor sizes across adenohypophyseal cell lineages (PIT-1, SF-1, T-PIT, null-cell, and high-risk tumors). Differences in sex and tumor histotypes among grades of the final grading system were evaluated using the χ^2^ test. Rank’s correlation coefficient assessed relationships between tumor grading, histotypes, and volumes.

#### Radiomics analysis for the final grading system score

Kruskal–Wallis test evaluated associations between radiomic features extracted from CE-T1-weighted and T2-weighted images and the final grading categories (1a, 1b, 2a, 2b). Benjamini–Hochberg correction adjusted for multiple comparisons. Pearson correlation (*r* > 0.7) removed redundant features. A *post hoc* Wilcoxon–Mann–Whitney test for invasive tumors assessed whether radiomic features distinguished tumor grades 2a and 2b to detect proliferative tumors.

#### Radiomic feature selection for invasiveness

Feature selection performed on the training set identified relevant, non-redundant radiomic features for invasiveness model development in each analysis (CE-T1-weighted, T2-weighted, CE-T1-weighted + T2-weighted). Wilcoxon–Mann–Whitney test discarded vendor-sensitive features (*p* < 0.05). Univariate analysis *via* the Wilcoxon–Mann–Whitney test retained features significantly associated with the binary outcome of invasiveness (adjusted *p* < 0.05). Pearson correlation (*r* ≥ 0.7) removed redundant features. Backward elimination was performed by removing the least important feature iteratively based on the average accuracy of a 5-fold cross-validation approach with logistic regression, random forest and support vector machine classifiers [42]. This allowed for reduced features, minimizing overfitting.

#### Radiomics modeling

A grid search optimized model hyperparameters on the training set using 5-fold cross-validation for model development. Model performance was assessed *via* the area under the curve (AUC) of the receiver operating characteristic (ROC) analysis. For each analysis (CE-T1-weighted, T2-weighted, CE-T1-weighted + T2-weighted), the best machine learning model was chosen based on AUC from the test set. ROC curves for training and test sets were compared to assess model generalizability. The models’ discriminative ability was evaluated using AUC, sensitivity, specificity, and accuracy with 95% confidence intervals calculated *via* bootstrap for AUC and normal approximation for other metrics.

## Results

### Patients’ characteristics

This study included 200 patients. Patient and tumor characteristics were summarized in Table 1.

**Table 1:** Demographic and clinical characteristics of patients with PitNETs and final Trouillas grading categorization

		Patients (*n*)
Median [IQR] age (years)	55 [47–66]	200
Sex	F	94
	M	106
Final grading system score (F %)	1a	74 (35.16%)
	1b	21 (76.19%)
	2a	70 (18.57%)
	2b	35 (62.85%)
	3	0 (0%)

*F* Functioning tumors, *PitNETs* Pituitary neuroendocrine tumors

Combining histological and radiological evaluation, 41.16% of tumors initially classified as noninvasive (based on radiology) were reclassified as grade 1b (15.44%), grade 2a (16.17%), or grade 2b (9.55%). Conversely, 34.37% of tumors classified as invasive (based on radiology) were reclassified as grade 2b (invasive and proliferative).

**Fig. 1:**
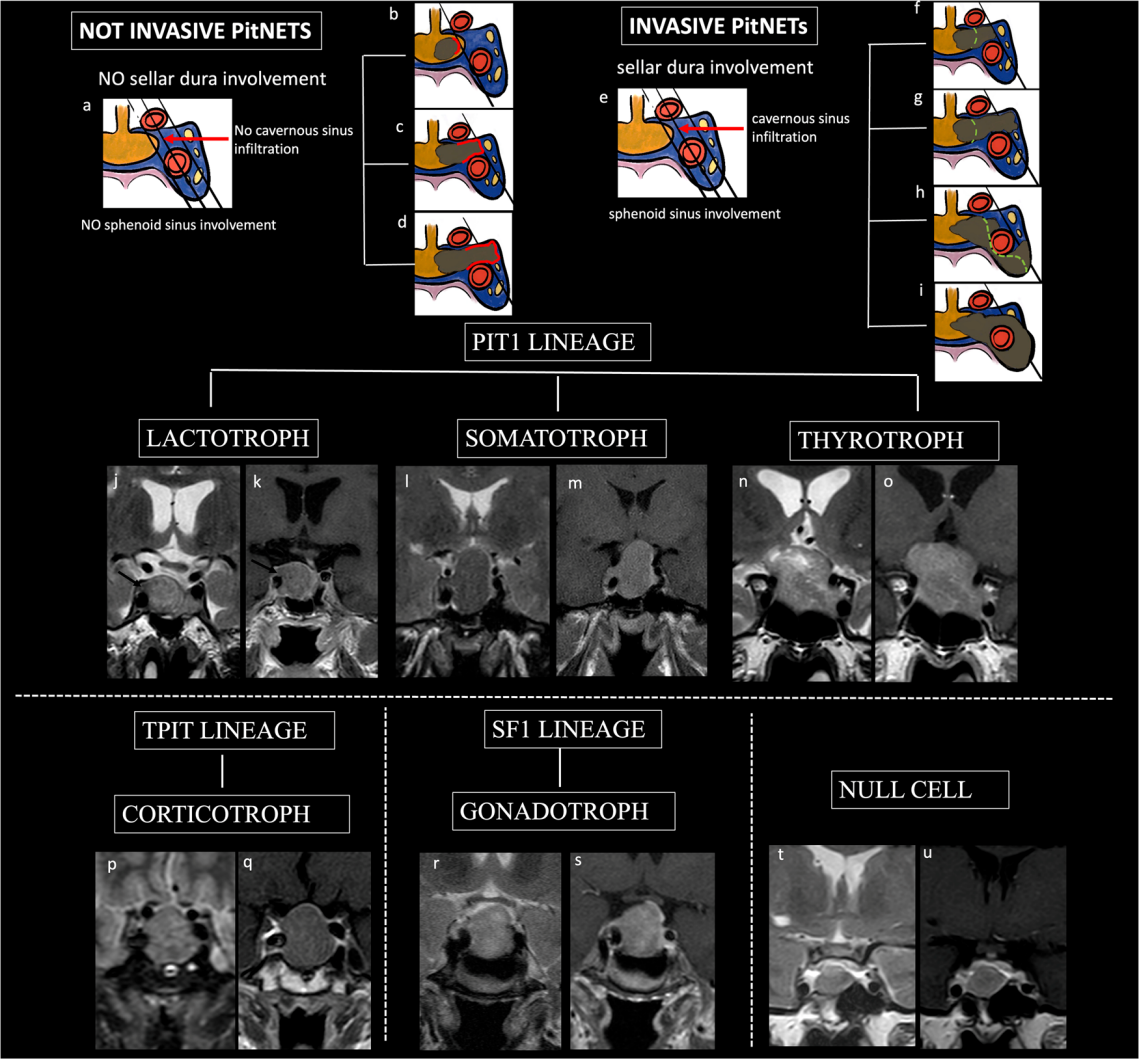
Classification of pituitary neuroendocrine tumors (PitNETs) (**a**, **e**) based on radiological-surgical invasiveness, morphofunctional histotypes, and cellular lineages. Coronal T2-weighted images (T2w) (**j**, **l**, **n**, **p**, **r**, **t**); coronal contrast-enhanced T1-weighted images (T1w) (**k**, **m**, **o**, **q**, **s**, **u**). Three lines (medial, median, and lateral) crossing the internal carotid artery (ICA) suggest the degree of radiological invasion of the medial dural wall of the cavernous sinus, but confirmation of invasiveness is achieved through surgical and histopathological assessment. Noninvasive PitNETs (**b**–**d**) do not infiltrate the sphenoid sinus, sella dura, or cavernous sinus dura (grades 1, 2, and 3a according to the modified Knosp classification). Knosp grade 1: PitNET reaching the medial line but not the median line, referred to as the intercarotid line (**b**). Knosp grade 2: PitNET reaching the lateral ICA aspects with intact medial cavernous sinus wall (**c**). Knosp grade 3a: PitNET extending beyond the lateral aspects of the ICAs and into the superior cavernous sinus compartment, yet with a widened but intact medial dural wall (**d**). Invasive PitNETs (**f**–**i**) infiltrate the dura mater of the sella, sphenoid sinus, and cavernous sinus dura (grades 2, 3a, 3b, or 4 according to the modified Knosp classification). Knosp grade 2: PitNET reaches lateral ICA aspects with medial cavernous sinus wall invasion (**f**). Knosp grade 3a: PitNET extending beyond the lateral aspects of the ICAs and into the superior cavernous sinus compartment, with interruption of the medial dural wall (**g**). Knosp 3b: PitNET extending beyond the lateral aspects of the ICAs and into the inferior cavernous sinus compartment, with disruption of the medial dural wall (**h**). Knosp 4: PitNET encasing the intercavernous ICA (**i**). According to pituitary cell lineages, pituitary adenoma may be: PIT-1 lactotroph PitNETs (**j**, **k**); somatotroph PitNET (**l**, **m**); thyrotroph PitNET (**n**, **o**); T-PIT corticotroph PitNETs (**p**, **q**); SF-1 gonadotroph PitNETs (**r**, **s**); null-cell PitNET (**t**, **u**)

No difference was observed among tumor grades for sex (*p* > 0.05), but age differed significantly, with older patients having non-proliferative tumors (*p* < 0.001). Tumor volume differed by histotype (*p* < 0.001), with “high-risk” and SF1 tumors being larger. SF1 tumors were more common in grades 1a and 2a (non-proliferative), while PIT-1 and “high-risk” tumors were more frequent in grades 1b and 2b (proliferative). Tumor volume was smaller in lower grades (*p* < 0.001) (Fig. 1).

### Radiomic feature extraction

A total of 204 features (102 from CE-T1-weighted and 102 from T2-weighted images) were extracted per subject: 1. morphological features: *n* = 14; 2. first-order features: *n* = 18; 3. tumor texture features: *n* = 70, subdivided into: GLCM: *n* = 24; GLRLM: *n* = 16; GLSZM: *n* = 16; GLDM: *n* = 14.

### Feature selection

#### Radiomics analysis for final grading system score

**Fig. 2:**
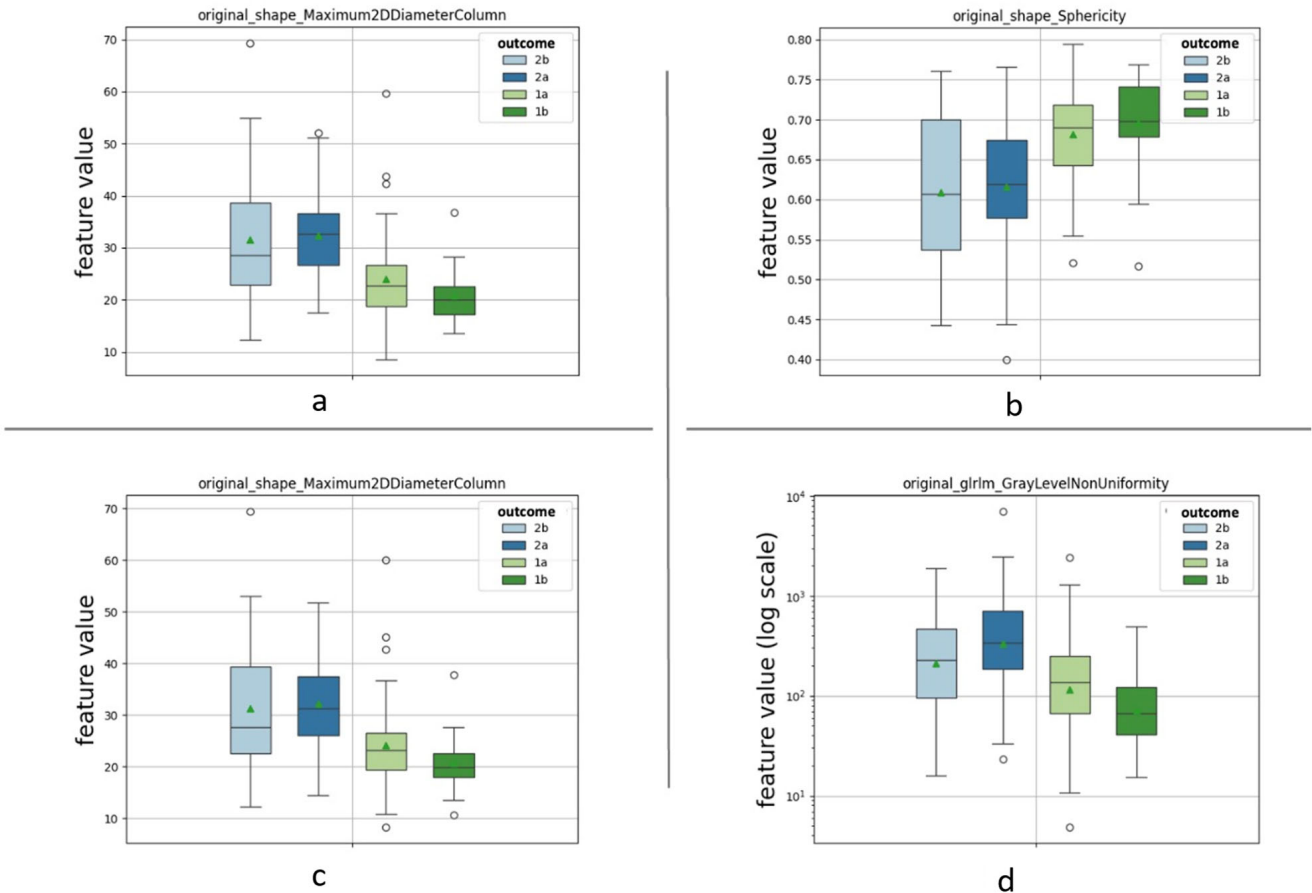
Boxplots of four representative statistically significant features associated with the final grading system score (1a, 1b, 2a, 2b) for contrast-enhanced T1-weighted (CE-T1w) preliminary analysis (**a**, **b**), and T2-weighted (T2w) preliminary analysis (**c**, **d**). These include morphological (shape) and texture (GLRLM) features

For the final grading classification, 73 features from CE-T1-weighted and 65 features from T2-weighted images were significant (adjusted *p* < 0.05). Of these, 13 uncorrelated features were identified for each image type. These features belong to all feature families (morphological-shape, first-order statistical, texture) (Supplementary Table S2). The four most statistically significant features associated with the final grading system for CE-T1-weighted and T2-weighted analyses belonged to the morphological (shape) and textural families (Fig. 2). No statistically significant feature was found between the two classes of invasive tumors (2a and 2b) in the *post hoc* analysis.

#### Radiomics analysis of invasiveness

**Fig. 3:**
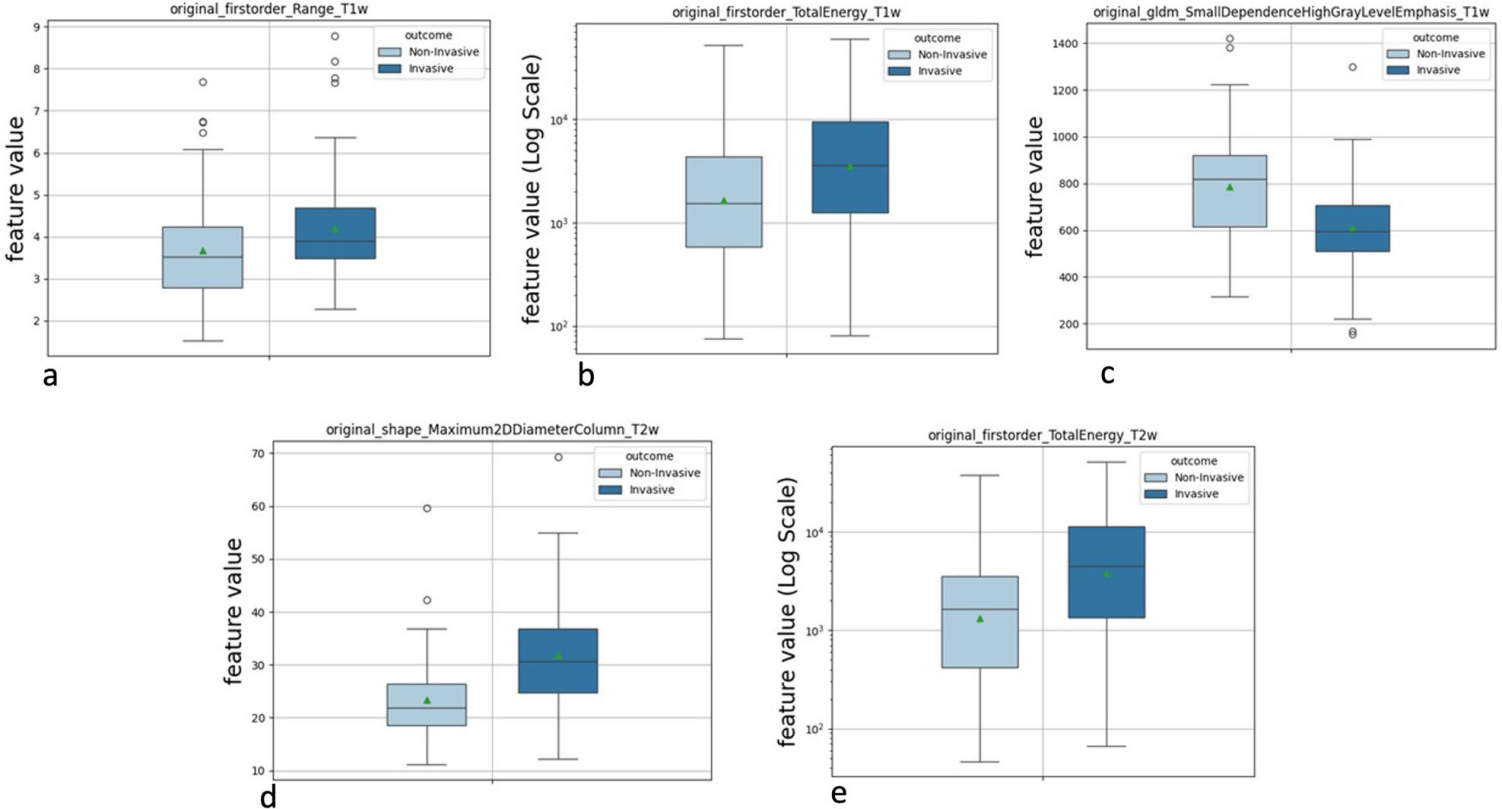
Boxplots of the five features obtained from the selection process of the combined CE-T1-weighted (**a**–**c**) and T2-weighted (**d**, **e**) analysis, used as inputs for the best-performing radiomic model. These include morphological (shape), first-order statistical (first-order) and texture (GLDM) features

For the binary outcome analysis (invasive *versus* noninvasive), feature selection yielded different numbers of features for each analysis: 5 for CE-T1-weighted, 6 for T2-weighted, and 5 for combined CE-T1-weighted + T2-weighted. Specifically, CE-T1-weighted analysis yielded two shape-related features (Sphericity, maximum 2D diameter), two textural-related features (gray-level non-uniformity from GLRLM, small dependence high gray-level emphasis from GLDM), and one first-order feature (range); T2-weighted analysis yielded two shape-related features (Sphericity, maximum 2D diameter), two textural-related features (run percentage from GLRLM, large area low gray-level emphasis from GLSZM) and two first-order features (range, total energy); CE-T1-weighted + T2-weighted analysis yielded one shape-related features (maximum 2D diameter), one textural-related feature (small dependence high gray-level emphasis from GLDM) and three first-order features (range and total energy) from both CE-T1-weighted and T2-weighted images (Table 2, Fig. 3). These features were used for radiomics modeling.

**Table 2:** Features used for the development of radiomics models obtained for each of the three different analyses (CE-T1-weighted, T2-weighted, CE-T1-weighted + T2-weighted)

CE-T1-weighted		T2-weighted		CE-T1-weighted + T2-weighted	
Feature	*p*-value	Feature	*p*-value	Feature	*p*-value
shape_Sphericity	7.44E-07	shape_Maximum2D DiameterColumn	2.60E-07	shape_Maximum2D DiameterColumn_T2w	4.06E-07
shape_Maximum2D DiameterColumn	7.44E-07	shape_Sphericity	2.90E-07	gldm_Small DependenceHighGray LevelEmphasis_T1w	6.84E-06
glrlm_GrayLevelNon Uniformity	6.10E-06	firstorder_Range	2.90E-05	firstorder_ TotalEnergy_T2w	1.15E-04
gldm_Smal lDependenceHighGrayLevelEmphasis	6.72E-06	firstorder_ TotalEnergy	1.23E-04	firstorder_ TotalEnergy_T1w	3.64E-03
firstorder_Range	5.33E-03	glrlm_RunPercentage	7.52E-03	firstorder_Range_T1w	6.06E-03
		glszm_LargeAreaLow GrayLevelEmphasis	1.92E-02		

The features used for the development of radiomics models belong to all radiomic feature families (morphological—shape, first-order statistical, texture—gldm, glszm, glrlm)

Adjusted statistically significant *p*-values derived from the Wilcoxon–Mann–Whitney test for the selected features are reported (*p*)

### Models performance

The training set comprised 75% (*n* = 150) and the test set 25% (*n* = 50) of the patients, with a balanced proportion of invasive cases (53% training, 52% test) and noninvasive cases (47% training, 48% set).

**Fig. 4:**
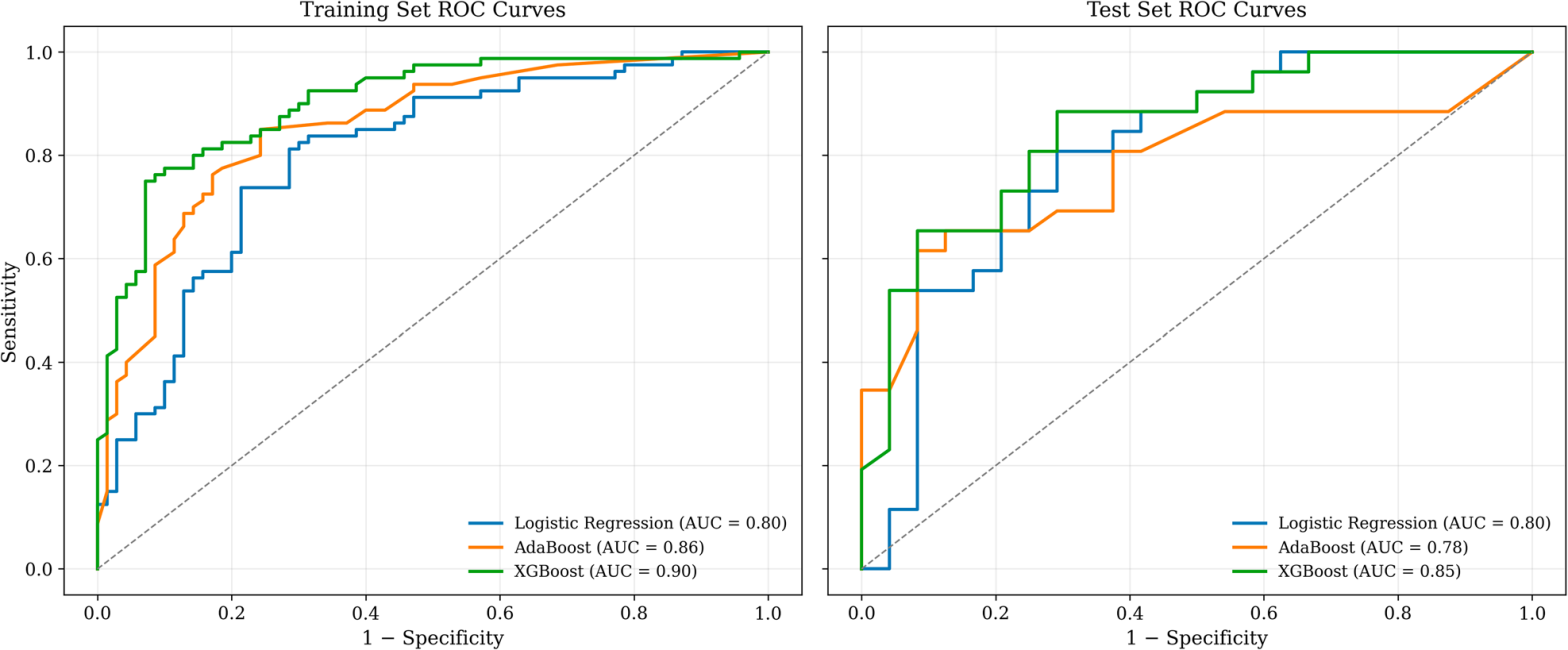
Receiver operating characteristic curves for the training set and the test set of the best model selected for each of the three analyses: logistic regression for CE-T1-weighted images (blue line), AdaBoost for T2-weighted images (orange line), and XGBoost for CE-T1-weighted + T2-weighted images (green line)

For each analysis (CE-T1-weighted, T2-weighted, CE-T1-weighted + T2-weighted), the best model was chosen based on the AUC in the test set. ROC curve analyses confirmed no overfitting and good generalizability (Fig. 4).

ML model performances, including AUC, accuracy, sensitivity and specificity, are reported in Table 3. The XGBoost model using features from both CE-T1-weighted and T2-weighted images outperformed logistic regression and AdaBoost models developed using a single sequence (CE-T1-weighted and T2-weighted, respectively). For the test set, the XGBoost model achieved an AUC of 0.85 (95% confidence interval: 0.75–0.95) and sensitivity of 0.81 (95% confidence interval: 0.66–0.96), comparable to the training set (Table 4). The confusion matrix associated with the performance metrics of the latter model is reported in the Supplementary Material (Supplementary Fig. S1).

**Table 3:** Performance obtained on the test set for the best model selected for each of the three conducted analyses (CE-T1-weighted, T2-weighted, CE-T1-weighted + T2-weighted)

Test set performance			
	CE-T1-weighted	T2-weighted	CE-T1-weighted + T2-weighted
ML model	Logistic regression	AdaBoost	XGBoost
AUC	0.80	0.78	0.85
Accuracy	0.74	0.66	0.76
Sensitivity	0.81	0.69	0.81
Specificity	0.67	0.62	0.71

**Table 4:** Training and test set performances for the XGBoost model developed with the combination of CE-T1-weighted and T2-weighted-based radiomic features

	XGBoost model CE-T1-weighted and T2-weighted	
	Training set	Test set
AUC	0.90 (95% CI: 0.85–0.94)	0.85 (95% CI: 0.75–0.95)
Accuracy	0.81 (95% CI: 0.74–0.91)	0.76 (95% CI: 0.64–0.88)
Sensitivity	0.82 (95% CI: 0.71–0.89)	0.81 (95% CI: 0.66–0.96)
Specificity	0.80 (95% CI: 0.75–0.88)	0.71 (95% CI: 0.53–0.89)

*AUC* Area under the ROC curve, *CI* Confidence interval

## Discussion

The primary aim of our study was to classify PitNETs using Trouillas criteria, including high-risk PitNETs within the proliferative category. Some tumors previously classified as noninvasive based on radiological findings were reclassified as grade 1b (noninvasive but proliferative), 2a (invasive but not proliferative), or 2b (invasive and proliferative). Conversely, some tumors initially classified as invasive through radiological assessment were reclassified as grade 2b. This confirms the importance of a multidisciplinary evaluation incorporating endocrinological, surgical, and histological data to achieve a correct classification [16].

The percentage of hormone-secreting tumors was higher in proliferative classes (grades 1b and 2b) than in non-proliferative classes (grades 1a and 2a). The distribution of tumor subtypes within each grade was also variable, with SF1 PitNETs more frequent in grades 1a and 2a, and PIT-1 and high-risk tumors more common in grades 1b and 2b.

Tumor grade correlated with volume, while histotype did not, suggesting that volume increases with grade, while histotype is unrelated to grade or invasiveness [43]. However, certain high-risk PitNETs with proliferative characteristics may require close monitoring due to recurrence risks [44, 45].

Radiomics quantifies pixel patterns in medical images, extracting numerical features beyond human visual detection [20, 21, 46]. However, few studies have applied radiomics to predict the PitNETs’ clinicopathological classification.

The second aim of our study was to assess the association between MRI-based radiomic features and final tumor grade using Trouillas’ classification system. Features were extracted from the whole tumor using T2-weighted and CE-T1-weighted images [47, 48]. These sequences were effective in quantifying tumor heterogeneity [49, 50]. Various studies showed that factors such as collagen content, intra-tumoral hematoma, amyloid, iron deposits, calcification, and protein-rich fluid can influence T2 signal intensity [51]. Meanwhile, CE-T1-weighted, which reflects blood-brain barrier damage, may better delineate tumor boundaries and shape, providing an accurate assessment of adjacent tissue invasion [52–54].

We identified two representative shape-related features (sphericity, maximum 2D diameter) from CE-T1-weighted images, and one shape-related feature (maximum 2D diameter) along with a texture-related feature (GLRLM) from T2 images as the most statistically significant in stratifying PitNETs into the four categories of Trouillas’ final grading system. Tumor sphericity and maximum diameter shape features reflect PitNETs’ growth pattern [55, 56]. Meanwhile, the GLRLM texture feature assesses tumor gray-level heterogeneity [27, 28, 49]. Specifically, higher tumor grades (2a and 2b) showed lower values of sphericity, higher values of maximum diameter, and greater tissue gray-level heterogeneity, confirming that higher-grade tumors are more irregular and heterogeneous, and more likely to invade surrounding tissues.

No significant differences among radiomic features were found in the two invasive tumor classes (2a and 2b), so we combined the grades of the final Trouillas’ system into two categories: noninvasive (grades 1a and 1b) and invasive (grades 2a and 2b). The last goal of our study was to develop machine learning (ML) models to predict the invasiveness of solid or microcystic PitNETs. We developed an image pre-processing pipeline to achieve optimal radiomic feature evaluation and classification analysis into the two invasiveness categories. Three ML radiomic models were identified as the best models, utilizing features from the CE-T1-weighted, T2-weighted, and combined CE-T1-weighted-T2-weighted sequences.

The XGBoost model, incorporating features from both CE-T1-weighted and T2-weighted images, outperformed the logistic regression and AdaBoost models using a single sequence (either CE-T1-weighted or T2-weighted), showing strong generalizability with consistent performance across training and test sets.

Our data support previous reports affirming that multi-modal imaging outperforms single-modal imaging in diagnostic accuracy [57]. The radiomic features that enhanced the performance of the predictive XGBoost model included one shape-related feature from T2-weighted (shape-maximum 2D diameter), one texture-related feature from CE-T1-weighted (GLDM), and three first-order features, of which one is from T2-weighted (total energy) and two are from CE-T1-weighted (range and total energy). This finding suggests that combining texture, shape, and first-order features from CE-T1-weighted and T2-weighted images—which capture information on size, distribution, spatial inter-relationships of gray signal intensity values, and variations in gray-level intensity—offers a more accurate representation of overall tumor growth pattern and heterogeneity, thereby enhancing the prediction of tumor invasiveness. In particular, invasive PitNETs are those with a greater maximum diameter, lower compactness, higher gray signal intensity values, and a more rapid increase in variations in gray-level intensity, reflecting more heterogeneous tumors [56, 58].

Limitations of this proof-of-concept study include the retrospective nature of this single-center study and manual tumor segmentation, which can introduce inconsistencies. The lack of assessment of interobserver variability, such as the Intraclass correlation coefficient or Dice coefficient, prevents us from drawing conclusions about the consistency and agreement between observers. Although we used coronal pre-contrast T2-weighted and CE-T1-weighted images—commonly referred to in clinical practice—to build the proposed radiomics predictive model, incorporating other sequences such as diffusion-weighted imaging might provide additional information and improve model performance. Although a hold-out set was used to assess the model’s performance on unseen data, using multiple splits or nested cross-validation would ensure model stability, while an external test set from another center would be the best approach to demonstrate model generalizability. A future multicenter study with external validation is necessary to confirm our model’s effectiveness. Lastly, the development and integration of automatic tumor segmentation methods based on deep-learning models in the future will enable the practical applicability of such a predictive radiomic model into clinical practice.

In conclusion, this study provides new insights into PitNETs’ stratification using a radiomic approach aligned with the Trouillas classification system. Tumor shape and texture features are associated with the classification of PitNETs into the four grades of the Trouillas system, with lower sphericity, larger diameter, and increased heterogeneity identifying invasive tumors (2a and 2b).

The ML XGBoost model, integrating tumor shape, texture, and first-order features from combined CE-T1-weighted and T2-weighted images, demonstrates high discriminative ability in predicting PitNET invasiveness. This noninvasive approach could help identify tumors with invasive growth patterns, optimizing individualized treatment strategies before surgery.

## Supplementary information

**Additional file 1: Supplementary Table S1.** CLEAR checklist with explanations. **Supplementary Table S2**. Uncorrelated features that resulted statistically significant with respect to the final grading system score. **Supplementary Figure S1.** Confusion matrix relative to performance metrics reported for the XGBoost model developed on the CE-T1w+T2w sequence.

## Data Availability

The data supporting the findings of this study are available from the corresponding author upon reasonable request.

## Abbreviations

2D: Two-dimensional
AUC: Area under the receiver operating characteristic curve
CE: Contrast-enhanced
GLDM: Gray-level dependence matrix
GLRLM: Gray-level run-length matrix
GLSZM: Gray-level size zone matrix
ML: Machine learning
PIT: Pituitary‑specific positive transcription factor
PitNETs: Pituitary neuroendocrine tumors
ROC: Receiver operating characteristic
SF-1: Steroidogenic factor-1
T-PIT: T‑box pituitary transcription factor (TBX19)-PIT

## Code availability

The codes supporting the findings of this study are available from the corresponding author upon reasonable request.
